# Pigs as Source of Methicillin-Resistant *Staphylococcus aureus* CC398 Infections in Humans, Denmark

**DOI:** 10.3201/eid1409.071576

**Published:** 2008-09

**Authors:** Hannah C. Lewis, Kåre Mølbak, Catrin Reese, Frank M. Aarestrup, Mette Selchau, Marit Sørum, Robert L. Skov

**Affiliations:** Statens Serum Institut, Copenhagen, Denmark (H.C. Lewis, K. Mølbak, M. Selchau, M. Sørum, R.L. Skov); European Programme for Intervention Epidemiology Training (H.C. Lewis); Vejle Hospital, Vejle, Denmark (C. Reese); Technical University of Denmark, Copenhagen (F.M. Aarestrup)

**Keywords:** Staphylococcus aureus, methicillin resistance, community-acquired infections, epidemiology, case-control studies, animals, domestic swine (pig), CC398, research

## Abstract

An emerging subtype of methicillin-resistant *Staphylococcus aureus* (MRSA), clonal complex (CC) 398, is associated with animals, particularly pigs. We conducted a matched case–control and a case–case study comparing 21 CC398 case-patients with 2 controls randomly selected from the Danish Civil Registry and 2 case-patients infected with MRSA other than CC398. On farms of case-patients, animals were examined for MRSA. Thirteen case-patients reported pig exposure. Living or working on farms with animals was an independent risk factor for CC398 in the case–control (matched odds ratio [MOR] 35.4, 95% confidence interval [CI] 2.7–469.8) and the case–case study (MOR 14.5, 95%CI 2.7–76.7). History of hospitalization was associated with an increased risk only in the case–control study (MOR 11.4, 95% CI 1.4–94.8). A total of 23 of 50 pigs on 4 of 5 farms were positive for CC398. Our results, corroborated by microbiologic testing, demonstrate that pigs are a source of CC398 in Denmark.

## Abstract 2

Persons living or working on farms, particularly pig farms, are at increased risk for infection.

Methicillin-resistant *Stapylococcus aureus* (MRSA) is becoming increasingly recognized among persons in the community without established risk factors (*1*,*2*). MRSA primarily causes human disease and animals have not, until now, been considered a source of infection.

It has recently become apparent that animals, particularly pigs, can constitute a separate MRSA reservoir and be a source of a novel and rapidly emerging type of MRSA in humans; namely MRSA clonal complex (CC)398 by multilocus sequence typing (MLST) (*3*). MRSA CC398 consists of 8 MLST types (www.saureus.mlst.net), the predominant type being sequence type (ST)398, and a range of closely related protein A (*spa*) gene types (i.e., t011, t034, t108, and t1793) (*4*,*5*).

Although transmission appears to be primarily between animals, indistinguishable isolates have been found in their human contacts, particularly those with occupational exposure (*3*–*7*). MRSA CC398 (ST398) was first detected in 4 pigs and 1 healthy pig farmer in France (*3*,*8*). Clinical infection was described in the daughter of a pig farmer in the Netherlands in 2004 (*7*). That study showed that 23% of pig farmers in a small survey in the same region were seropositive for MRSA CC398.

Denmark has a low incidence of MRSA. In 2006, only 706 new MRSA patients (colonization only or infection) were reported, which corresponds to 13/100,000 population (*9*). To maintain this status, Denmark has adopted a strict “Search and Destroy” policy, which includes active screening of at-risk persons at admission to a hospital (*10*). Prompted by reports from the Netherlands, we identified a small (<1% of all MRSA patients) but increasing number of MRSA CC398 human patients after 2003. Furthermore, CC398 was detected in a pig in Denmark in 2006 (*11*). With an annual production of ≈25 million slaughter pigs (www.dst.dk), Denmark has a large potential reservoir for MRSA CC398.

We report results of an analytical study of MRSA CC398, in parallel with systematic farm and microbiologic investigations, to identify risk factors for MRSA CC398 acquisition in persons in Denmark. Although farm and pig exposure have been postulated as risk factors after interviews with MRSA CC398 patients in other studies (*4*,*6*,*7*,*12*), no previous study has included interviews with control populations to determine if these exposures were higher than would be expected in the general population.

## Methods

### Surveillance

In Denmark, MRSA isolates from all human patients have been referred to Statens Serum Institut since 1988 for characterization and national surveillance. Epidemiologic and clinical information has been obtained prospectively since 1999 on all patients.

### Case-Patients and Controls

We conducted a matched case–control study comparing human case-patients with MRSA CC398 during 2004–2007 with 2 population controls. In parallel, we conducted a case–case study comparing the same MRSA CC398 case-patients to 2 case-patients of community-detected MRSA of a type other than CC398 (non-CC398 case-patients). Eligible case-patients were persons with a confirmed diagnosis of MRSA with *spa* types related to CC398 (carriage or infection) during the study period: October 29, 2003 (first human diagnosed) to May 31, 2007. Where household clusters were identified, secondary case-patients were excluded from the study. Population controls were selected randomly from the Danish Civil Registry System and matched by sex, date of birth, and residence in the same municipality. Non-CC398 case-patients were selected from the national MRSA database and matched by sex, residence in the same region (Zealand, Jutland, or Funen), age group (± 10 years for adults and ± 3 years for persons <18 years of age) according to whether infected or a carrier, and similar time of diagnosis (90% ± 4 months) to limit differential recall bias. When >2 non-CC398 MRSA case-patients were identified within ± 4 months of diagnosis of the case-patient, 2 were randomly selected from the list generated.

### Data Collection

After written informed consent was obtained, case-patients, controls, and non-CC398 case-patients were interviewed by using a structured telephone-administered questionnaire. Questions captured demographic and clinical data as well as information on known/identified risk factors for MRSA (including hospitalization and other medical exposures, contact sports, and travel) and hypothesized farm and animal exposures for MRSA CC398 (including living or working on a farm, exposure to farm animals, contact with farm workers, preference for eating, and contact with pets) in the year before case-patient diagnosis. Data were double-entered into Epidata version 3.1 (Statens Serum Institut, Copenhagen, Denmark) and collected and handled according to the requirements of the Danish Data Protection Agency. The study did not require ethical approval.

### Statistical Analysis

Univariable and multivariable conditional logistic regression analyses were conducted to estimate matched odds ratios (MORs). Unmatched logistic regression was used for variables for which MORs could not be calculated because not enough controls were exposed to MRSA. Multivariable conditional logistic regression analysis included significant variables (based on a p value of 0.05) from univariable analysis. Stepwise exclusion was used, and variables were tested for significance by using the likelihood ratio test. Stata version 9.2 software (StataCorp, College Station, TX, USA) was used for all analyses.

### Farm Investigations

For case-patients, controls, and non-CC398 case-patients reporting contact with production animals, the farm owner was contacted. If the owner consented, nasal swabs were taken from 10 randomly selected animals (from 10 different pens where possible) and tested for MRSA.

### Microbiologic Analysis

Human isolates were tested by using PCR to identify the *mecA* gene (*13*), pulsed-field gel electrophoresis (PFGE) with the Harmony protocol (*14*), *spa* typing (*15*), and staphylococcal cassette chromosome mec (SCC*mec*) typing (*16*). Isolates were also tested for *lukF/lukS* genes encoding Panton-Valentine leukocidin (PVL) (*17*). Results of PFGE and *spa* typing were interpreted by using BioNumerics version 4.6 software (Applied Maths, Sint-Martens-Latem, Belgium). Because *spa* typing is acknowledged as being a proxy for MLST, the MLST clonal complex annotation was inferred on the basis of *spa* types. One of the human isolates was typed by MLST for confirmation. Furthermore, a random selection of 7 isolates were tested by PCR for the exotoxin genes *tst*, *eta*, and *etb* encoding staphylococcal toxic shock syndrome toxin 1, exfoliative toxin A, and exfoliative toxin B, respectively (*18*).

Animal swabs were plated directly on CHROM-MRSA agar (Becton Dickinson, Heidelberg, Germany) and blood agar (containing 5% bovine blood) and subsequently placed in selective broth (tryptic soy broth, 2.5% salt, aztreonam [20 mg/L], and cefoxitin [3.5 mg/L]; SSI Diagnostika, Hillerød, Denmark). After incubation for 24 hours, the CHROM-MRSA agar was inspected for putative MRSA. Subcultivation on CHROM-MRSA was conducted with samples from the enrichment broth. Possible MRSA colonies were subcultivated on blood agar plates, identified by PCR for the *mecA gene* (*13*), and subjected to *spa* typing and SCC*mec* typing.

Human and animal isolates underwent antimicrobial drug susceptibility testing by using disc diffusion (D) with Neosensitabs (Rosco, Taastrup, Denmark) on Danish blood agar (SSI Diagnostika) or microbroth dilution (M) (*19*). Susceptibility tests were performed for tetracycline (D and M), erythromycin (D and M), streptomycin (D and M), kanamycin (D), norfloxacin (D), pencillin (D), clindamycin (D), fusidic acid (D), rifampicin (D), cefoxitin (D), ceftiofur (M), chloramphenicol (M), ciprofloxacin (M), florfenicol (M), spectinomycin (M), sulfamethoxazole (M), tiamulin (M), and trimethoprim (M).

## Results

### Descriptive Epidemiology

Thirty-one case-patients with MRSA with *spa* types related to MRSA CC398 were detected from October 29, 2003, through February 16, 2007. Of these, 6 were excluded from the study because they were secondary case-patients (3 family clusters). Of the remaining eligible case-patients, we were unable to interview 4 because of a death (n = 1) and refusal to participate (n = 3). The questionnaire was therefore administered to 21 of 25 primary case-patients. Median age of the case-patients was 29 years (age range 8 months to 80 years), and 13 (62%) were female. Three case-patients reported having Dutch relatives, and 2 case-patients had a connection to the People’s Republic of China; 1 case-patient was an adopted child from China and another case-patient had adopted a child from China.

Ten case-patients (48%) reported having had an infection, of which all were skin and soft tissue infections. Moreover, sinusitis developed in 1 case-patient, and a severe invasive infection with multiorgan failure after knee surgery developed in another case-patient.

### Univariable Analysis

Several exposure variables related to farms and animals were associated with CC398 in the case–control and case–case studies (Table 1). Case–control analysis also identified 4 medical-related risk factors (Table 1). No association was found in the case–control and case–case studies for the following exposures: travel 12 months before diagnosis, working in the healthcare sector, contact with primary healthcare sector (doctor, specialist), visiting an emergency department, presence of a person in the household with a skin condition, presence of a person in the household with staphylococcal infection, smoking daily, contact sports, owning or having contact with dog(s) or horse(s), preference for eating pork, and being born outside Denmark.

**Table 1:** Statistically significant associations by univariable analysis for infection with MRSA CC398, Denmark*

Exposure variable	No. (%) case-patients exposed (n = 21)		Odds ratio (95% confidence interval) for MRSA infection	
			Case–control study, population controls (n = 42)	Case–case study, non-CC398 case-patients (n = 39)
Animal and farm-related exposures				
Lived or worked on farm with animals	14 (67)		22.1 (2.9–170.3)	11.6 (2.6–51.7)
Worked with animals or meat	11 (50)		16.2 (2.0–127.8)	∞†
Worked on farm with animals	10 (48)		∞†	∞†
Lived on farm with animals	9 (43)		6.9 (1.5–32.8)	7.9 (1.7–36.7)
Exposure to pigs	13 (62)		∞†	∞†
Exposure to cattle	6 (29)		∞†	∞†
Exposure to other farm animals (hens, goats, sh eep)	7 (33)		11.1 (1.4–92.4)	5.9 (1.2–28.8)
Provided antimicrobial drugs to animals	10 (48)		∞†	∞†
Contact with farm workers	16 (76)		5.2 (1.4–19.3)	∞†
Contact with farmer	14 (67)		3.2 (1.0–10.6)	∞†
Contact with veterinarian	7 (33)		6.3 (1.3–30.7)	6.6 (1.4–31.8)
Lived in countryside	13 (62)		7.2 (1.5–33.8)	5.2 (1.4–18.9)
Had cat in home	11 (50)		3.2 (1.0–10.6)	3.4 (1.1–9.9)
Used manure in garden	7 (33)		3.2 (0.9–11.0)	6.6 (1.4–31.8)
Visited farm, zoo, or stables	12 (57)		0.8 (0.3–2.4)	4.7 (1.3–17.4)
Medical-related exposures				
Admission to hospital in 12 mo before diagnosis	13 (62)		6.8 (1.9–24.4)	1.5 (0.4–5.2)
Someone in household with chronic condition	12 (57)		3.8 (1.2–12.5)	2.0 (0.8–9.3)
Antimicrobial drug use in 12 mo before diagnosis	9 (43)		3.4 (1.0–11.5)	2.6 (0.8–9.2)
Contact with person with skin sore or other skin infection	5 (26)		8.6 (1–74.9)	0.6 (0.2–1.9)

*MRSA, methicillin-resistant S*taphylococcus aureus*; CC, clonal complex. †p<0.01, by unmatched analysis.

### Multivariable Analysis

Logistic regression models were applied separately to the case–control and case–case studies. In both studies, the first model only included farm and animal-related exposures: lived or worked on a farm with animals, worked with animals or meat, exposed to pigs, exposed to cattle, exposed to other farm animals, provided antimicrobial drugs to animals, owned cat(s), and had contact with any farm workers. Other farm and animal exposures were excluded because of colinearity. In both case–control and case–case studies, living or working on a farm with animals remained the independent association in this first model. A second model combined living or working on a farm with animals with medical-related exposures: contact with a person having a skin sore or other skin infection, history of hospital admission in the 12 months before diagnosis, antimicrobial drug use in the 12 months before diagnosis, and someone in the household with a chronic condition.

Living or working on a farm with animals was an independent risk factor for CC398 in the case–control study (MOR 35.4, 95% confidence interval [CI] 2.7–469.8) and the case–case study (MOR 14.5, 95% CI 2.7–76.7). A history of hospital admission in the 12 months before diagnosis was associated with an increased risk in the case–control study (MOR 11.4, 95% CI 1.4–94.8) but not in the case–case study.

### Farm Investigations

Nine pig farms and 2 cattle herds, with which 10 case-patients had contact, were identified. One case-patient had contact with 2 pig farms, and 2 case-patients had contact with the same pig farm. No controls or non-CC398 case-patients had direct contact with a pig or cattle farm. The owners of 5 pig farms and of the 2 cattle herds agreed to participate in the study. The length of time between date of patient diagnosis and farm sampling was 2–24 months.

### Microbiologic Analysis

All but 1 of 31 human isolates were nontypeable by PFGE after digestion with *Sma*I. Twenty-nine isolates had *spa* type t034, including the isolate typeable by PFGE, and the other 2 were related variants of t034 (t108 and t1793). Because of the strong correlation between *spa* typing and MLST, all isolates could be assigned to CC398. One isolate (PFGE nontypeable, *spa* type t034) was typed by MLST and confirmed to be ST398. SCC*mec* typing showed that 24 isolates harbored SCC*mec* type V. SCC*mec* type IV was also found in 2 isolates (PFGE nontypeable, *spa* type t034). Three isolates were *ccr*AB2 positive, which indicated either a type II or type IV variant, but the *mec* class could not be determined. Two isolates were nontypeable. Isolates from the 21 case-patients were *spa* types t034 (SSCmec IV, n = 2, SSCmec V, n = 16, and a type II or type IV variant, n = 1, typeable by PFGE), t108 (SSCmec V, n = 1), and t1793 (SSCmec V, n = 1). These isolates showed considerable variation in antimicrobial drug resistance patterns; most isolates were resistant to tetracycline and erythromycin. All isolates from case-patients who reported exposure to pigs were tetracycline resistant and PVL negative. Two isolates were PVL positive; these were from case-patients who reported a connection to China. All isolates tested for exotoxin showed negative results for all toxins examined.

Twenty-three (46%) of 50 pigs on 4 of 5 sampled farms carried CC398 *spa* type t034. All isolates were resistant to tetracycline and trimethoprim. Pig isolates were indistinguishable or only differed by 1 additional antimicrobial drug class when compared with isolates from case-patients who had contact with them (Table 2). MRSA was not detected in the 2 cattle herds.

**Table 2:** Isolate characteristics for human case-patients and contact pig herds sampled, Denmark, March 2007*

Case-patient no.	Date of diagnosis	Resistance pattern	*spa* type	Contact herd	No. isolates	Resistance pattern	*spa* type
1	2005 Mar	tet, ery, cli, str, tmp	t034	A	9	tet, ery, cli, str, spe, tmp	t034
2	2005 Oct	tet, tmp	t034	B	5	tet, tmp	t034
					4	tet, str, tmp	t034
					1	tet, str, tmp	t038
3	2006 Oct	tet, ery, cli, str, spe, tmp	t034	C	1	tet, ery, cli,str, spe, tmp	t034
					2	tet, str, spe, tmp	t034
4	2006 Nov	tet, kan, str, spe, tmp	t034	D	1	tet, ery, cli, kan, str, spe, tmp	t034

*spa, staphylococcal protein A; tet, tetracycline; ery, erythromycin; cli, clindamycin; str, streptomycin; tmp, trimethoprim; spe, spectinomycin; kan, kananmycin.

## Discussion

This study provides compelling epidemiologic and microbiologic evidence that persons living or working on farms in Denmark, particularly pig farms, are at increased risk of being colonized or infected with MRSA CC398. We provide evidence for pigs being a substantial reservoir of human MRSA CC398 in Denmark, as appears to be the case in other European countries such as the Netherlands and France (*3*,*4*,*7*,*12*), and in Canada (*6*).

In the case–case analysis, only animal and farm-related exposures were associated with being a case-patient, which indicates that these exposures are the major factors associated with CC398 acquisition. Furthermore, comparison of results from the case–control study (where farm/animal and medical-related variables remained associated) indicates that medical-related exposures are risk determinants for community-detected MRSA in general but not specifically for subtype CC398. This finding can be deduced because the design of the case–case analysis controls for exposures common to both groups, which means that these exposures will not be identified as a risk or might be underestimated (*20*).

Because of evidence of prolonged MRSA carriage (*21*), questions related to exposures referred to a period of 1 year before patient diagnosis. This lengthy recall period is a limitation of this study. However, all questions related to memorable activities and lifestyle choices; any bias introduced is therefore thought to be minimal.

Our finding that living or working on a farm with animals is associated with CC398 acquisition reinforces results of studies in France, the Netherlands, and Canada that indicated that CC398 is transmissible from animals to humans (*3*,*4*,*6*,*7*). Also in the Netherlands, a CC398 prevalence of 3.9% in 179 veterinarians has been described; all positive persons had recent or regular contact with pigs and cows (*22*). Screening participants from 38 countries at a veterinary conference in Denmark in 2006 found a CC398 prevalence of 9.6% (26/272); the highest prevalence was in German and Dutch delegates (*23*). In comparison, the prevalence of CC398 among delegates attending several national animal conferences in Denmark was 0.7% (4/576) (*24*).

Pig isolates from contact herds were indistinguishable, or only differed by 1 additional antimicrobial drug class, from the isolates from human case-patients who worked or lived on farms where these pigs were located. These samples were obtained months, in some cases years, after the human case-patient’s infection was diagnosed, findings that lend weight to the hypotheses that CC398 carriage in animals is unlikely to be transient and that animals are a reservoir of CC398. Although we isolated CC398 from 23 pigs on 4 of 5 pig farms, but not from cattle farms, this type may not be exclusive to pigs. CC398 has been shown to have a prevalence of 39% in pigs in the Netherlands (*25*). However, it has also been found in horses and dogs in Germany and Austria (5) and in cattle and poultry in the Netherlands (*26*,*27*). These findings are particularly interesting because *S*. *aureus* is usually host specific (*28*).

Our study has highlighted other important human epidemiologic aspects of CC398. The clinical picture for CC398, with ≈50% of case-patients having had an infection and all reporting skin and soft tissue infections, is similar to that seen in community-acquired MRSA isolates in general (*29*,*30*). There was a serious invasive infection with MRSA reported in a man in Denmark after surgery on his knee; arthritis and multiorgan failure also developed (*31*). Serious complications from CC398 have also been described in other reports including ventilator-associated pneumonia (*5*) and endocarditis (*32*). Because no statistical association was found with travel abroad, this finding indicates that CC398 is endemic among pigs in Denmark. Nevertheless, there was an overrepresentation of case-patients or their family members who have had contact with another country, 2 with adopted children from China and 3 case-patients with relatives from the Netherlands. Likewise, in the Netherlands, a child adopted from China was found to be an MRSA CC398 carrier in 2004 (*12*). Three family clusters were identified in the present study, which indicates that CC398 can be transmitted from person to person. This finding is not surprising because of the adaptability of MRSA. Its potential for transmission between humans has also been observed in the Netherlands (*4*,*7*). MRSA has been isolated from dairy products, beef, chicken and pork (*33*–*37*) and although foodborne transmission is plausible (*34*), the risk is thought to be low. Preference for eating pork was not associated with being a CC398 case-patient in our study.

A high degree of variability in the types of CC398 (resistance patterns, *spa* types, and SCC*mec* types) suggests that this type is either rapidly evolving or emerging from a hitherto unrecognized reservoir. In the latter case, CC398 must have been introduced into Denmark on more than 1 occasion or by various routes to explain the high degree of variance. When one considers the rapid adaptability of MRSA, it may only be a matter of time before we see an increased prevalence of CC398 in humans, including those in hospitals as has been recently reported in the Netherlands (*12*). A high prevalence of tetracycline resistance in CC398 patients in contact with pigs has also been observed in the Netherlands; this finding suggests that use of tetracyclines and possibly other antimicrobial drugs in food animals is selecting these multidrug-resistant bacteria (*25*). Two case-patients who were positive for PVL had direct connections to China. To our knowledge, there are no published reports of CC398 patients in China but isolates from pigs have recently been reported from Singapore, with Indonesian origin (*38*).

In conclusion, transmission of CC398 from a zoonotic reservoir to humans could undermine existing MRSA control programs. We therefore recommend increased awareness among healthcare professionals that animals are a possible source of MRSA infection and that the potential for person-to-person spread exists. To limit further spread, pig farmers may warrant screening and isolation on admission to hospitals as has been implemented in the Netherlands (*39*). However, further studies are required to better understand the human and veterinary epidemiology of this emerging zoonosis. Areas of study should include size of the reservoir in pigs, whether other animals constitute a reservoir of CC398, and how frequently CC398 is transmitted from animals to humans and from humans to humans. The European Union baseline survey on the prevalence of MRSA in breeding pigs, initiated in January 2008, is an important step in addressing the first of these points (*40*).
